# Clinical strategy of diagnosing and following patients with nonalcoholic fatty liver disease based on invasive and noninvasive methods

**DOI:** 10.1007/s00535-017-1414-2

**Published:** 2017-11-24

**Authors:** Masato Yoneda, Kento Imajo, Hirokazu Takahashi, Yuji Ogawa, Yuichiro Eguchi, Yoshio Sumida, Masashi Yoneda, Miwa Kawanaka, Satoru Saito, Katsutoshi Tokushige, Atsushi Nakajima

**Affiliations:** 1 Department of Gastroenterology and Hepatology, Yokohama City University Graduate School of Medicine, 3-9 Fukuura, Kanazawa-ku, Yokohama, 236-0004 Japan; 2 Division of Metabolism and Endocrinology, Faculty of Medicine, Saga University, 5-1-1 Nabeshima, Saga, 849-8501 Japan; 3 Liver Center, Saga University, 5-1-1 Nabeshima, Saga, 849-8501 Japan; 4 Division of Hepatology and Pancreatology, Department of Internal Medicine, Aichi Medical University School of Medicine, 1-1 Yazakokarimata, Nagakute, Aichi 480-1195 Japan; 5 General Internal Medicine 2, General Medical Center, Kawasaki Medical School, 2-6-1 Nakasange, Kutaku, Okayama, 700-8505 Japan; 6 Department of Internal Medicine and Gastroenterology, Tokyo Women’s Medical University, 8-1 Kawada-cho, Shinjuku-ku, Tokyo, 162-8666 Japan

**Keywords:** Nonalcoholic fatty liver disease, Scoring system, Elastography, Liver biopsy, MR elastography

## Abstract

Nonalcoholic fatty liver disease (NAFLD) is an important cause of chronic liver injury in many countries. The incidence of NAFLD is rising rapidly in both adults and children, because of the currently ongoing epidemics of obesity and type 2 diabetes. Notably, histological liver fibrosis is recognized as the main predictive factor for the overall long-term outcome of NAFLD, including cardiovascular disease and liver-related mortality. Thus, staging of liver fibrosis is essential in determining the prognosis and optimal treatment for patients with NAFLD and in guiding surveillance for the development of hepatocellular carcinoma (HCC). Whereas liver biopsy remains the gold standard for staging liver fibrosis, it is impossible to enforce liver biopsy in all patients with NAFLD. Noninvasive biological markers, scoring systems and noninvasive modalities are increasingly being developed and investigated to evaluate fibrosis stage of NAFLD patients. This review will highlight recent studies on the diagnosis and staging of NAFLD based on invasive (liver biopsy) or noninvasive (biomarker, scoring systems, US-based elastography and MR elastography) methods.

## Introduction

Nonalcoholic fatty liver disease (NAFLD) is an important cause of chronic liver injury in many countries [1, 2]. NAFLD ranges from benign nonalcoholic fatty liver (NAFL) to nonalcoholic steatohepatitis (NASH). The latter condition includes progressive fibrosis, which is associated with high rates of overall and disease-specific mortality [3], hepatocellular carcinoma (HCC) [4, 5] and atherosclerotic diseases [6]. Liver biopsy is recommended as the gold standard method for the diagnosis of NASH and the staging of liver fibrosis in patients with NASH [7]. However, because of its increased cost, risk and healthcare resource use, an invasive liver biopsy is a poorly suited diagnostic test for such a prevalent condition [8]. Therefore, the development of reliable noninvasive methods for the assessment of liver fibrosis has become essential to estimate the potential progression of NASH to cirrhosis and HCC and to guide therapy. In this review, we highlight recent advances in biomarkers, scoring systems, ultrasound (US)-based elastography techniques and magnetic resonance imaging (MRI) techniques with which to evaluate the liver fibrosis stage and steatosis grade and discuss their usefulness for surveillance of the liver status, including the presence or absence of HCC, in patients with NAFLD.

## Liver biopsy

Histological analysis of liver biopsy samples has played a central role in the management of NAFLD and NASH in terms of diagnosis, definition of severity, and prediction of prognosis. Hepatic steatosis is a histological hallmark of NAFLD. Hepatic steatosis independent from alcohol consumption in morbid obesity and diabetes had been reported in the decades after 1952 [9–11] and Ludwig et al. finally propounded NASH in 1980 [12]. Studies in the 1970s identified that most fatty liver did not progress to fibrosis and cirrhosis [13, 14]. Therefore, hepatic steastosis was considered as a benign or non-harmful findings; however, it was unknown whether the other concomitant findings including lobular inflammation, Mallory–Denk bodies, ballooning degeneration and fibrosis were associated with the disease progression of NAFLD/NASH.

In 1999, Matteoni et al. confirmed the progressive course of patients who had NAFLD with ballooning degeneration [15]. As a result of these differences, these authors classified NAFLD with hepatic steatosis and ballooning degeneration as type 3 and NAFLD with liver steatosis, ballooning degeneration, fibrosis, and Mallory–Denk bodies as type 4 (Table 1). Types 3 and 4 NAFLD are associated with higher liver-related mortality rates than type 1 (only liver steatosis) and type 2 (liver steatosis and lobular inflammation) [15]. NAFLD can be divided into NAFL without ballooning degeneration and NASH with ballooning degeneration [16, 17]. Matteoni’s classification, which is based on analysis of the liver-related prognosis, has been used as the standard classification system for the diagnosis of NASH. However, this study and classification by Matteoni et al. left three main issues for other researchers to resolve over the next decade: a too-small sample size of patients with type 2 and 3 NAFLD (*n* = 10 and 19, respectively) to confirm the impact of ballooning degeneration on prognosis, intraobserver and interobserver differences in the diagnosis of ballooning degeneration, and a missing classification for the disease severity of NAFLD [18, 19]. The classification published by Brunt et al. in 1999 enabled definition of the severity of NASH as an activity grade (grades 1–3) and fibrosis stage (stages 1–4) [20] (Table 1). The NAFLD activity score (NAS) was developed by the NASH Clinical Research Network Pathology Committee based on Brunt’s classification and is mainly used to judge treatment responses or disease progression in clinical studies [21] (Table 1).

**Table 1:** Pathological classification of NAFLD for diagnosis and evaluation of disease severity

References	Type	Description	Diagnosis of NASH
Matteoni [15]	Diagnosis	Type 1 (steatosis) Type 2 (steatosis + lobular inflammation) Type 3 (steatosis + ballooning) Type 4 (steatosis + fibrosis or steatosis + MDB)	Type 3 and 4
Brunt [20]	Severity	Grade (activity) 1–3 Stage (fibrosis) 1–4^a^	Not for diagnosis
NAFLD activity score (NAS) [21]	Diagnosis	Steatosis 0–3 Inflammation 0–3 Ballooning 0–3	Total score is 5 (4) or higher than 5 (4)
Younossi [23]	Diagnosis	(1) Steatosis + centrilobular ballooning and/or MDB (2) Steatosis + centrilobular pericellular/perisinusoidal fibrosis or bridging fibrosis	(1) or (2)

*MDB* Mallory–Denk bodies

^a^ Stage 1 was particularly classified by Kleiner et al. as a part of NAS (delicate perisinusoidal fibrosis in the perivenular area as 1A, dense perisinusoidal fibrosis in the perivenular area as 1B and detection of portal fibrosis without perisinusoidal fibrosis was defined as 1C.)

The modified diagnostic criteria for NASH published by Younossi et al. in 2011 were a turning point in the diagnosis of NASH (Table 1). In their studies, NASH was diagnosed in the presence of: (1) any degree of steatosis along with centrilobular ballooning and/or Mallory–Denk bodies or (2) any degree of steatosis along with centrilobular pericellular/perisinusoidal fibrosis or bridging fibrosis in the absence of another identifiable cause [22, 23]. The most important difference from Matteoni’s classification is the significance of ballooning degeneration and fibrosis; according to the modified criteria, NASH can be diagnosed without ballooning degeneration if fibrosis is identified. Younossi et al. also performed a multivariate analysis of prognosis, and liver fibrosis was the only histological finding independently associated with liver-related mortality [23]. This result confirmed that liver fibrosis in patients with NASH is more important than ballooning degeneration in terms of the liver-related prognosis of NASH. In a multi-center study performed by Angulo et al. [24] in 2015, the severity of liver fibrosis was correlated with the hazard ratio of the overall morality or receipt of liver transplantation (hazard ratio of 1.92 in patients with stage 3 fibrosis and 6.35 in those with stage 4 fibrosis compared with patients without fibrosis). Dulai et al. recently performed a meta-analysis with similar results with respect to an overall mortality rate that increased with progression of the fibrosis stage [25]. In terms of liver-related mortality, they also found that the mortality rate ratio increased exponentially as the fibrosis stage increased compared with patients without fibrosis (1.4 in patients with stage 1 fibrosis, 9.6 in stage 2, 16.7 in stage 3, and 42.3 in stage 4) [25].

Consequently, liver fibrosis could be considered the most clinically important histological finding of NAFLD. Another advantage of focusing on liver fibrosis is its high reliability. In addition, the widespread general use of noninvasive alternatives to histological evaluation of liver fibrosis, including vibration-controlled transient elastography (VCTE™, FibroScan; Echosens, Paris, France) and blood biomarkers, might paradoxically prove the importance of histological liver fibrosis again in future clinical studies.

Efforts to diagnose NASH or non-NASH using histological findings other than liver steatosis and fibrosis now seem to be less significant in the context of long-term clinical outcomes; however, whether improvement of liver fibrosis can contribute to prolonged survival and decreased liver mortality remains unknown. On the other side, the latest updated practice guidance from the American Association for the Study of Liver Diseases (AASLD) reconfirmed the importance of ballooning degeneration, lobular inflammation, and Mallory–Denk bodies for diagnosing NASH [26]. The histological findings that are associated with better outcomes should be confirmed because they might represent a treatment target in the era of developing new agents for NAFLD.

## Biomarkers and scoring systems

**Fig. 1:**
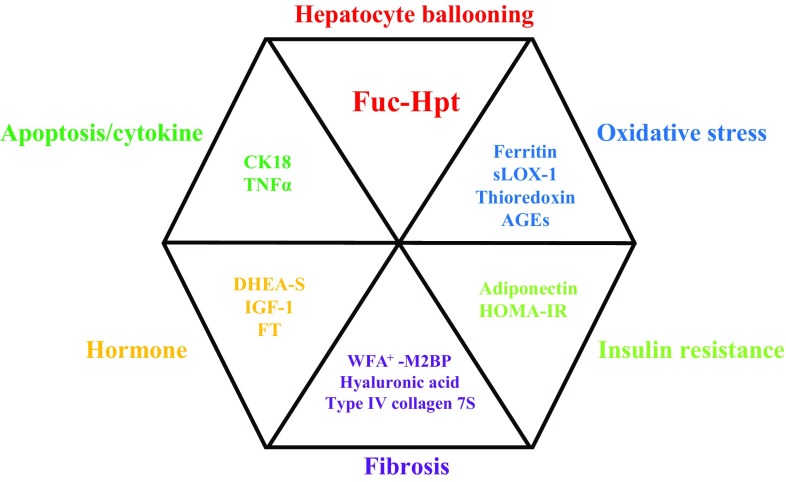
Noninvasive biomarkers for the diagnosis of nonalcoholic steatohepatitis.* Fuc-Hpt* Fucosylated haptoglobin, *CK18* cytokeratin-18 fragment,* TNFα* tumor necrosis factor-α,* DHEA-S* dehydroepiandrosterone sulfate,* IGF-1* insulin-like growth factor-1,* FT* free testosterone,* sLOX-1* oxidized low-density lipoprotein receptor-1,* AGEs* advanced glycation end products,* HOMA-IR* homeostasis model assessment-insulin resistance,* WFA+ -M2BP* wisteria floribunda agglutinin mac-2-binding protein

As previously stated, liver biopsy is defined the gold standard for the diagnosis of NASH. However, the pathological diagnosis of NASH has several limitations such as interobserver variability, absence of diagnostic consensus, and sampling error. Various noninvasive biomarkers have been proposed to detect NASH for avoiding redundant liver biopsies. These biomarkers include indicators of insulin resistance (homeostatic model assessment-insulin resistance [HOMA-IR], adiponectin), oxidative stress (thioredoxin, advanced glycation end products [AGEs]), inflammation (high-sensitivity C-reactive protein, tumor necrosis factor-α), and apoptosis (cytokeratin-18 fragment) as well as hormones (insulin-like growth factor 1, dehydroepiandrosterone sulfate, and free testosterone) and hepatic fibrosis markers (Fig. 1). Platelet (PLT) count is the simplest index for predicting advanced fibrosis in patients with NAFLD [27]. Notably, PLT levels may be unexpectedly high even when hepatic fibrosis is advanced (18.9 × 10^4^/μL in patients with stage 3 NASH). Fibrosis markers also have been extensively examined, including hyaluronic acid, type IV collagen 7S, procollagen III peptide and *Wisteria floribunda* agglutinin-positive Mac-2-binding protein (WFA+ -M2BP). A recent meta-analysis showed that measurement of the serum level of WFA+ -M2BP, which is now covered by the health insurance system in Japan, is useful for detecting severe fibrosis or predicting HCC development in patients with chronic liver disease [28]. WFA+ -M2BP is also of clinical use for assessing liver fibrosis in patients with NAFLD [29, 30]. Plasma collagen type III, which is a neo-epitope marker that reflects true type III collagen formation [31], is a useful test with which to predict fibrogenesis and monitor disease progression [32]. Recently, Kamada et al. demonstrated that serum levels of fucosylated haptoglobin can reflect histological hepatocyte ballooning in NAFLD-affected liver [33, 34]. However, these fibrosis markers have some drawbacks such as high cost, difficulty of generalizability in clinical practice, and lack of sufficient evidence among different ethnicities.

Numerous non-invasive panels of tests have been developed for the staging of liver disease consisting of combinations of clinical and routine laboratory parameters, as well as specialized tests. The aspartate aminotransferase-to-alanine aminotransferase ratio (AAR) is also a simple index with which to detect advanced fibrosis, although it has limitations when used alone [35]. It is accepted that patients with NAFLD who have an AAR of > 0.8 are likely to have advanced fibrosis, as observed in the BARD score (which comprises a body mass index [BMI] of > 28 kg/m^2^ [= point], AAR of > 0.8, and the presence of diabetes) [36]. The NAFLD fibrosis score (NFS) [37] and the fibrosis-4 (FIB-4) [38] index have been externally validated in populations of different ethnicities with consistent results [39]. These scores have been proven useful for not only excluding advanced fibrosis (stage ≥ 3) but also predicting liver-related mortality [40, 41]. The combination of the PLT count (< 15.3 × 10^4^/μL), albumin concentration (< 4.0 g/dL), and AAR (> 0.9; i.e., the PLALA score) is a good predictor of cirrhosis in patients with NAFLD based on data from the Japan Study Group of NAFLD (JSG-NAFLD) group [42]. Surprisingly, the cut-off serum albumin level was higher than expected. The serum albumin level should be monitored to avoid misdiagnosis of cirrhosis in patients with NAFLD. A multicenter study by the JSG-NAFLD showed that measurement of the serum ferritin, fasting insulin, and type IV collagen 7S, the combination of which is called the NAFIC score, is the most useful method with which to detect NASH according to the data of 619 Japanese patients with biopsy-proven NAFLD [43]. Okanoue et al. recently suggested that the combination of type IV collagen 7S and aspartate aminotransferase (CA index-NASH = 0.994 × type IV collagen 7S + 0.0255 × aspartate aminotransferase) is a reliable and simple scoring system with which to differentiate NASH from NAFLD. Patients with NAFLD with a CA index-NASH of > 7 are likely to have NASH [44]. These scoring systems from Japan (NAFIC score and CA index) should be validated in an independent population.

## Genetic Factors that affect NAFLD

Genetic factors are important for the development of NAFLD, as well as environmental factors. Recent genome-wide association studies revealed that genetic variation, rs738409 (I148M), in the patatin-like phospholipase domain-containing protein 3 (*PNPLA3*) on chromosome 22 influences NAFLD [45]. It is associated with NASH also in Japanese patients with NAFLD [46, 47]. In one study, the prevalence of the *PNPLA* genotypes GG, CG, and CC was 20, 50, and 30%, respectively, in the Japanese general population [48]. Interestingly, the *PNPLA3* G allele is more prevalent in Japanese than Caucasian individuals [48, 49]. In addition, *PNPLA3* GG homozygosity is strongly associated with hepatocarcinogenesis in patients with NAFLD [49, 50].

Transmembrane 6 superfamily 2 (*TM6SF2*) has broad tissue and organ expression with highest relative levels of expression in the small intestine and liver [51]. The chromosomal location of the *TM6SF2* gene in human is 19p13.11. One variant in *TM6SF2* (rs58542926, E167K) is positively associated with elevations in serum AST and ALT [52]. Exome-wide association study (EWAS) identified a variant of this gene that is associated with NAFLD [52]. The *TM6SF2* rs58542926 variant was strongly associated with NAFD, advanced fibrosis, and cirrhosis, independent of age, BMI, type 2 DM and *PNPLA3* [53]. Both the *PNPLA3* and the *TM6SF2* have been examined in recent phase 2 and phase 3 clinical trials. In the near future, it is expected that determination of *PNPLA3* and *TM6SF2* single-nucleotide polymorphisms will be available in daily clinical practice.

## Ultrasound-based elastography

Conventional US imaging plays a major role in the diagnosis of chronic liver disease and is routinely used for this purpose. Furthermore, US is the most common technique for evaluation of hepatic steatosis because of its availability, lack of radiation exposure, and low cost. The mean sensitivity of US for identification of steatosis ranges from 73.3 to 90.5% [54]. The sensitivity of US for detecting mild steatosis (0–10% on liver biopsy) is relatively lower. A decreased steatosis grade is observed with progression to advanced liver fibrosis, especially cirrhosis in patients with NAFLD [55]. In addition, conventional US cannot accurately differentiate the various stages of liver fibrosis in patients with NAFLD. Therefore, conventional US is not able to discriminate among steatosis, fibrosis, inflammation, and existence of NASH [56, 57] (Supplementary Table 1). To assess liver fibrosis, several noninvasive US-based elastography techniques have been developed. These methods include VCTE, acoustic radiation force impulse (ARFI) imaging and shear wave elastography (SWE). We herein discuss the principles, advantages, diagnostic accuracy, and limitations among different US-based elastography techniques for predicting liver fibrosis in patients with NAFLD.

### VCTE

US-based VCTE performed with the FibroScan (Echosens) is the most thoroughly validated and commonly used elastography method worldwide [58–70] (Table 2). VCTE is equipped with a one-dimensional probe and an ultrasonic transducer mounted on the axis of a vibrator. A vibration of mild amplitude and low frequency is transmitted from the vibrator onto the tissue by the transducer itself, which induces propagation of an elastic shear wave through the tissue [71]. The propagation velocity is directly related to the stiffness of the medium, defined by the Young modulus expressed in kilopascals (*E*≒3ρVs2) [71].

**Table 2:** Performance of liver stiffness measurement by transient elastography compared with liver biopsy in the detection of fibrosis in patients with NAFLD

Fibrosis stage	Cut-off value (kPa)	AUROC	Se	Sp	PPV	NPV	References
Stage ≥ 2	6.65	0.865	0.88	0.74	0.79	0.85	Yoneda M et al. Dig Liver Dis. 2008 [61]
Stage ≥ 3	9.8	0.904	0.85	0.81	0.64	0.93	
Stage = 4	17.0	0.991	1.00	0.97	0.75	1.00	
Stage ≥ 2	7.4	0.99	1	0.92	0.8	1	Nobili V et al. Hepatology. 2008 [65]
Stage ≥ 3	10.2	1.00	1.00	1.00	1.00	ND	
Stage ≥ 2	7	0.84	0.79	0.76	0.7	0.84	Wong VW et al. Hepatology. 2010 [66]
Stage ≥ 3	8.7	0.93	0.84	0.83	0.6	0.95	
Stage = 4	10.3	0.95	0.92	0.88	0.46	0.99	
Stage ≥ 2	6.8	0.79	0.67	0.84	0.6	0.88	Lupsor M, et al. J Gastrointestin Liver Dis. 2010 [67]
Stage ≥ 3	10.4	0.98	1	0.97	0.71	1	
Stage ≥ 2	7.25	0.795	0.69	0.7	ND	ND	Petta S et al. Aliment Pharmacol Ther. 2011 [58]
Stage ≥ 3	8.75	0.87	0.76	0.78	ND	ND	
Stage ≥ 2	7	ND	0.76	ND	ND	ND	Gaia S, et al. J Hepatol. 2011 [68]
Stage ≥ 3	8	ND	0.65	ND	ND	ND	
Stage = 4	10.5	ND	0.78	ND	ND	ND	
Stage > 2	7.0 (6.2)	0.83 (0.80)	0.79 (0.73)	0.64 (0.66)	0.62 (0.54)	0.80 (0.75)	Wong VW et al. Am J Gastroenterol. 2012 [59]
Stage ≥ 3	8.7 (7.2)	0.87 (0.85)	0.83 (0.78)	0.84 (0.78)	0.58 (0.60)	0.93 (0.89)	
Stage = 4	10.3 (7.9)	0.89 (0.91)	0.81 (0.88)	0.83 (0.76)	0.35 (0.35)	0.98 (0.98)	
Stage > 2	7.6	0.81	73	78	48	91	Naveau S et al. Obes Surg. 2014 [59]
Stage = 3	7.6	0.85	100	74	27	100	
Stage > 2	9.8 (6.2)	0.82	0.6 (0.9)	0.9 (0.45)	ND	ND	Cassinotto C et al. Hepatology 2016 [64]
Stage ≥ 3	12.5 (8.2)	0.86	0.57 (0.9)	0.9 (0.61)	ND	ND	
Stage = 4	16.1 (9.5)	0.87	0.65 (0.92)	0.9 (0.62)	ND	ND	
Stage > 2	11	0.82	0.65	0.89	0.88	0.66	Imajo K et al. Gastroenterology. 2016 [62]
Stage ≥ 3	11.4	0.88	0.86	0.84	0.75	0.92	
Stage = 4	14	0.92	1	0.76	0.73	1	
Stage > 2	6.9	0.86	0.79	0.82	0.66	0.9	Park CC et al. Gastroenterology. 2017 [63]
Stage ≥ 3	7.3	0.8	0.78	0.78	0.45	0.94	
Stage = 4	6.9	0.69	0.63	0.66	0.15	0.95	
Stage > 2	7.8	0.83	0.82	0.78	0.68	0.88	Chen J et al. Radiology 2017 [70]
Stage ≥ 3	7.6	0.84	0.84	0.64	0.43	0.92	
Stage = 4	14.6	0.9	0.82	0.92	0.64	0.97	

*AUROC* Area under the receiver-operating characteristic, *NPV* negative predictive value, *PPV* positive predictive value, *Se* sensitivity, *Sp* specificity

VCTE was developed approximately 10 years ago as the first US-based elastography method. It has since been validated for liver fibrosis assessment and was recently included in the European Association for the Study of the Liver Guidelines for fibrosis assessment in patients with chronic B and C hepatitis infection [72]. Furthermore, VCTE received approval from the United States Food and Drug Administration on 5 April 2013, and it is expected that its use will subsequently increase not only in Europe but also the USA.

A systematic review and meta-analysis of VCTE in patients with NAFLD by Kwok et al. indicated that VCTE is good for the diagnosis of stage 3 fibrosis (85% sensitivity and 82% specificity) and excellent for stage 4 (92% sensitivity and 92% sensitivity). However, it has a slightly lower accuracy for diagnosing stage 2 fibrosis (79% sensitivity and 75% specificity) [73]. In a prospective 4-year study by Suzuki et al., the disease progression in patients with NAFLD was evaluated using VCTE [74]. The authors concluded that liver stiffness measurement (LSM) may be clinically useful to monitor the severity of hepatic fibrosis in patients with NAFLD. Additional prospective studies regarding the monitoring of LSM progression in patients with NAFLD are expected.

The controlled attenuation parameter (CAP) is a novel technology for grading steatosis by measuring the degree of US attenuation by hepatic fat. A meta-analysis by Karlas et al. involving 2735 patients showed that the area under the receiver operating characteristic curve (AUROC) was 0.825 (optimal cut-off, 248 dB/m) and 0.865 (optimal cut-off, 268 dB/m) for those with grade > 0 and > 1 steatosis, respectively [75]. The CAP is thought to be clinically useful because its measurement is cost-effective, easy to perform, liver-specific, and reproducible within patients. CAP measurement can be easily repeated to monitor changes in steatosis. In addition, the CAP and LSM can be simultaneously measured using the FibroScan M and XL probes.

The benefits of VCTE include its rapidity and painlessness, quick availability of the result, high intra- and inter-operator reproducibility (intra-class correlation coefficient [ICC] of 0.98) [76], and good diagnostic accuracy that has been validated in many studies [58, 59, 61–70] (Table 3). Clinical use of VCTE has generally been limited because of its high failure rate or unreliable results. These limitations are commonly a result of obesity, operator inexperience, narrow intercostal spaces, a thick chest wall, and ascites [77, 78]. In a previously published study, the rate of failed and unreliable measurements by VCTE using the standard M probe was 18.9–29.2% [77, 78]. To improve this problem, the VCTE device has three different probes for measurement in various circumstances: the S probe (5.0 MHz) for children, the M probe (3.5 MHz) for adults, and the XL probe (2.5 MHz) for overweight patients. Furthermore, information regarding the visualized image of the studied area and the LSM results from the left liver lobe are unavailable.

**Table 3:** Performance of liver stiffness measurement by ARFI compared with liver biopsy in the detection of fibrosis in patients with NAFLD

Design	Fibrosis stage	Cut-off value (m/s)	AUROC	Se	Sp	PPV	NPV	References
Prospective, single center *N* = 54	Stage ≥ 3	1.77 m/s	0.973	1	0.91	0.71	1	Yoneda M et al. Radiology. 2010 [83]
	Stage = 4	1.9 m/s	0.976	1.00	0.96	0.75	1.00	
Prospective, single center *N* = 23	Stage ≥ 3	1.47 m/s	0.94	1	0.75	ND	ND	Osaki A et al. World J Gastroenterology. 2010 [85]
Prospective, single center *N* = 172	Stage ≥ 3	4.24 kPa (1.19 m/s)	0.90	0.9	0.9	ND	ND	Palmeri ML et al. J Hepatol. 2011 [82]
Prospective, single center *N* = 64	Stage ≥ 2	1.16 m/s	0.94	0.85	0.9	ND	ND	Fierbinteanu Braticeviti C et al. Ultrasound Med Biol. 2013 [84]
	Stage ≥ 3	1.48 m/s	0.98	0.86	0.95	ND	ND	
	Stage = 4	1.64 m/s	0.98	0.92	0.92	ND	ND	
Prospective, single center N = 125	Stage ≥ 2	1.34 m/s	0.848	0.82	0.78	0.57	0.92	Cui J et al. Hepatology 2016 [81]
	Stage ≥ 3	1.34 m/s	0.896	0.95	0.74	0.43	0.99	
	Stage = 4	2.48 m/s	0.862	0.78	0.93	0.47	0.98	
Prospective, multi-center *N* = 291	Stage ≥ 2	1.32 m/s	0.77	0.56	0.91	ND	ND	Cassinotto et al. Hepatology 2016 [64]
	Stage ≥ 3	1.53 m/s	0.84	0.59	0.9	ND	ND	
	Stage = 4	2.04 m/s	0.84	0.44	0.9	ND	ND	
Prospective, single center *N* = 97	Stage ≥ 2	1.18 m/s	0.86	0.78	0.88	0.85	0.82	Attia D et al. Aliment Pharmacol Ther. 2016 [80]
	Stage ≥ 3	1.47 m/s	0.96	0.94	0.97	0.94	0.97	
	Stage = 4	1.89 m/s	0.93	0.86	0.94	0.78	0.96	

*AUROC* Area under the receiver-operating characteristic, *NPV* negative predictive value, *PPV* positive predictive value, *Se* sensitivity, *Sp* specificity

### ARFI imaging

ARFI imaging is a new method for quantifying mechanical properties of tissue. An ARFI pulse generates short-duration acoustic impulses in a small region of interest that cause mechanical excitation of liver tissue [79]. This technique was developed by Siemens (Virtual Touch Quantification; Siemens Healthineers, Erlangen, Germany) and followed by Philips (ElastPQ; Philips, Amsterdam, the Netherlands) and Samsung (Smart-Shearwave elastography; Samsung Medison, Seoul, South Korea). ARFI imaging involves targeting an anatomic region to be examined for its elastic properties while performing real-time B-mode imaging. As the shear waves propagate, the US machine is used to monitor the shear wave propagation. The measured shear wave speed is expressed in m/s.

Although less thorough than VCTE, ARFI imaging has also intensively been investigated in patients with NAFLD [64, 80–85] (Table 3). A meta-analysis in patients with NAFLD by Liu et al. involving 7 studies (723 patients) indicated that ARFI elastography is good for diagnosing stage > 2 fibrosis (80.2% sensitivity and 85.2% specificity) [86]. In 2016, however, Cassinotto reported that ARFI elastography performed equally well for stages > 3 and 4 but not stage > 2 fibrosis [64]. In the same study, Cassinotto et al. also reported a failure rate of 0.7% and unreliable liver stiffness in 18.2% of patients.

The benefits of ARFI elastography include its rapidity and painlessness, quick availability of the result, good intra-operator coefficient (ICC = 0.9) and inter-operator coefficient (ICC = 0.81) [87], ability to perform the technique during standard US examinations of the liver, and good diagnostic accuracy that has been validated in several studies [64, 80–85]. Limitations of the use of ARFI elastography in patients with NAFLD are that special expertise is required and quantification of steatosis is difficult.

### SWE

SWE was introduced on a diagnostic imaging device called the Aixplorer (SuperSonic Imagine, Aix-en-Provence, France) [88]; this was followed by development of the Virtual Touch Image Quant (Siemens Healthcare, Erlangen, Germany), LOGIQ E9/S8 (GE Healthcare, Little Chalfont, UK), and Aplio™500 (Toshiba Medical Systems, Odawara, Japan). The principle underlying SWE involves the combination of a radiation force induced in tissues by focused ultrasonic beams and an US imaging sequence with a very high frame rate able to capture the propagation of resulting shear waves in real time. SWE provides a real-time, two-dimensional (2D) quantitative map of liver tissue elasticity under conventional B-mode imaging; stiffer tissues appear red and softer tissues appear blue [88, 89]. The region of interest can be adjusted in terms of size and location, and the liver stiffness is quantitatively analyzed and expressed as Young’s modulus (kPa).

Cassinotto et al. performed the only study to evaluate the use of SWE in patients with NAFLD in 2016 [64]. Overall, SWE had diagnostic accuracy similar to that of VCTE and better than that of ARFI elastography for detecting stage > 2 fibrosis in patients with NAFLD. LSM failures occurred in 15% of patients, whereas unreliable results occurred in 7.2%. Reliable results were obtained in approximately 90% of patients with a BMI of < 30 kg/m^2^ but in only 73% of patients with a BMI of ≥ 30 kg/m^2^. The benefits of SWE include its rapidity and painlessness, quick availability of the result, excellent intra-operator coefficient (ICC = 0.95) and good inter-operator coefficient of (ICC = 0.88) [90], and ability to perform the technique during standard US examinations of the liver as ARFI imaging. It also has the advantage of being able to explore a larger field of view by choosing the size of the region of interest. Limitation of the use of SWE in patients with NAFLD is that few studies have reported results because SWE is a recent technique. And the disability of quantification about steatosis as ARFI imaging is another limitation. Finally, LSM failures were more frequent in obese patients.

## Magnetic resonance elastography

Magnetic resonance elastography (MRE) is an MRI-based method for quantitative imaging of tissue stiffness. It is available from several manufacturers of MRI scanners as an option that includes special hardware and software. Quantitative stiffness images (elastograms) of the liver can be rapidly obtained during breath-hold acquisitions and can therefore be readily included in conventional liver MRI protocols [91]. This is performed using similar principles to VCTE, whereby propagating shear waves are generated and imaged using phase-contrast MRI, which includes oscillating motion-sensitizing gradients. Inter-observer agreement for liver fibrosis staging in patients with viral hepatitis B and C is reportedly almost perfect for both histopathology (ICC = 0.91; 95% confidence interval [CI], 0.86–0.94) and MRE (ICC = 0.99; 95% CI, 0.98–1.00), with significantly higher agreement for MRE [92].

### Diagnostic accuracy of MRE for liver fibrosis staging in patients with NAFLD

An increasing number of studies have shown MRE to be an accurate method for diagnosing and staging hepatic fibrosis in patients with NAFLD (Table 4). Among the studies using MRE, the AUROC for diagnosis of stages > 1, > 2, > 3, and 4 ranged from 0.772 to 0.860, 0.856 to 890, 0.870 to 0.981, and 0.882 to 0.993, respectively [62, 63, 81, 93–98]. The corresponding MRE liver stiffness cut-offs for mild fibrosis (stage > 1) or advanced fibrosis (stage > 3) ranged from 2.50 to 3.02 kPa or 2.99 to 4.80 kPa with a sensitivity of 44–75% or 33–91% and specificity of 77–91% or 80–94%, respectively. A more advanced version of three-dimensional (3D) MRE was used in a recent prospective study demonstrating that 3D-MRE at 40 Hz has the highest accuracy for diagnosis of advanced fibrosis (stage > 3) in patients with NAFLD, while both 2D- and 3D-MRE at 60 Hz (the standard shear-wave frequency) are also very accurate for diagnosis of advanced fibrosis in patients with NAFLD [95].

**Table 4:** Performance of liver stiffness measurement by magnetic resonance elastography compared with liver biopsy in the detection of fibrosis in patients with NAFLD

Design	MRI (Tesla)	Comparison with scoring system	Comparison with US elastography	Fibrosis stage	Cut-off value (kPa)	AUROC	Se	Sp	PPV	NPV	References
Retrospective, single center *(N* = 142)	1.5 T	Yes	No	Stage ≥ 3	4.15	0.954	0.85	0.93	ND	ND	Kim et al. Radiology. 2013 [94]
Cross-sectional prospective, single center (*N* = 117)	3.0 T	No	No	Stage ≥ 1	3.02	0.838	0.554	0.907	0.911	0.542	Loomba et al. Hepatology. 2014 [96]
				Stage ≥ 2	3.58	0.856	0.657	0.915	0.767	0.862	
				Stage ≥ 3	3.64	0.924	0.864	0.905	0.679	0.966	
				Stage 4	4.67	0.894	0.800	0.944	0.571	0.981	
Individual participant data pooled analysis (*N* = 232)	1.5 or 3.0 T	No	No	Stage ≥ 1	2.88	0.86	0.75	0.77	ND	ND	Singh et al. Eur Radiol. 2016 [97]
				Stage ≥ 2	3.54	0.87	0.79	0.81	ND	ND	
				Stage ≥ 3	3.77	0.90	0.83	0.86	ND	ND	
				Stage 4	4.09	0.91	0.88	0.87	ND	ND	
Cross-sectional prospective, single center (*N* = 142)	3.0 T	Yes	Yes vs. VCTE (M probe)	Stage ≥ 1	2.5	0.80	0.750	0.857	0.990	0.846	Imajo et al. Gastroenterology. 2016 [62]
				Stage ≥ 2	3.4	0.89	0.873	0.850	0.884	0.836	
				Stage ≥ 3	4.8	0.89	0.745	0.869	0.745	0.810	
				Stage 4	6.7	0.97	0.909	0.945	0.588	0.992	
Cross-sectional, single center (*N* = 125)	3.0 T	No	Yes vs. ARFI	Stage ≥ 1	2.99	0.799	0.583	0.906	0.894	0.615	Cui et al. Hepatology. 2016 [81]
				Stage ≥ 2	3.62	0.885	0.667	0.957	0.846	0.889	
				Stage ≥ 3	3.62	0.934	0.905	0.933	0.731	0.980	
				Stage 4	4.15	0.882	0.889	0.914	0.444	0.991	
Cross-sectional prospective, single center (*N* = 100)	3.0 T 2D (60 Hz) 3D (40 or 60 Hz)	No	No	Stage ≥ 1 (3D,40 Hz)	1.77	0.848	ND	ND	ND	ND	Loomba et al. Am J Gastroenterol. 2016 [95]
				Stage ≥ 2 (3D,40 Hz)	2.38	0.856	ND	ND	ND	ND	
				Stage ≥ 3 (3D,40 Hz)	2.43	0.981	1.000	0.937	0.722	1.000	
				Stage 4 (3D,40 Hz)	3.21	0.993	ND	ND	ND	ND	
Cross-sectional prospective, single-center (*N* = 104)	3.0 T	No	Yes vs. VCTE (M and XL probe)	Stage ≥ 1	2.65	0.82	0.765	0.791	0.813	0.739	Park et al. Gastroenterology. 2017 [63]
				Stage ≥ 2	2.86	0.89	0.793	0.818	0.657	0.898	
				Stage ≥ 3	2.99	0.87	0.778	0.803	0.483	0.938	
				Stage 4	3.35	0.87	0.750	0.814	0.273	0.972	
Cross-sectional prospective, multi-center (*N* = 90), Child	3.0 T	No	No	Stage ≥ 1	2.78	0.772	0.444	0.907	0.762	0.710	Schwimmer et al. Hepatology. 2017 [98]

*AUROC* Area under the receiver-operating characteristic, *NPV* negative predictive value, *PPV* positive predictive value, *Se* sensitivity, *Sp* specificity

### Comparison of diagnostic accuracy for liver fibrosis staging among scoring systems, US elastography, and MRE in patients with NAFLD

Several studies have demonstrated that MRE is superior to biomarkers, scoring systems, and US-based elastography for the diagnosis of liver fibrosis in patients with NAFLD. Cui et al. demonstrated that MRE is more accurate than ARFI imaging for diagnosing any type of fibrosis in patients with NAFLD, especially those who are obese [81]. Imajo et al. demonstrated that MRE has greater diagnostic performance in the detection of fibrosis in patients with NAFLD than VCTE using an M probe, scoring systems (aminotransferase-to-PLT ratio index, FIB-4 index, BARD score, and NFS) [62]. In addition, Park et al. found MRE to be more accurate than TE using both M and XL probes in identification of liver fibrosis (stage ≥ 1) [63].

### MRI proton density fat fraction

The proton density fat fraction (PDFF), which is the fraction of MRI-visible protons bound to fat divided by all protons in the liver (bound to fat and water), is an MRI-based method for quantitative assessment of hepatic steatosis and is available as an option from several manufacturers of MRI scanners. Chemical shift imaging is applied to separate the liver signal into its water and fat signal components by acquiring gradient echoes at appropriately spaced echo times. In some variants of this approach, only the magnitude data are retained while the phase data are discarded; these variants accurately quantify the hepatic PDFF from 0 to 50%, fortuitously capturing the biological range of human hepatic steatosis, which rarely exceeds 50% [99]. The precision and reproducibility of MRI-PDFF assessment have been further explored. Negrete et al. showed high inter-examiner agreement in obese subjects for each hepatic segment (ICC ≥ 0.992; standard deviation [SD], ≤ 0.66%; range, 0.00–1.24%), each lobe (ICC ≥ 0.998; SD, ≤ 0.34%; range, 0.00–0.64%), and the whole liver (ICC = 0.999; SD, ≤ 0.24%; range, 0.00–0.45%) [100]. Similarly excellent intra- and inter-examination precision in overweight and obese subjects was demonstrated by Tyagi et al. [101]. Bannas et al. further demonstrated significantly smaller variance with excellent intra-observer and inter-observer agreement and repeatability for MRI-PDFF compared with histologic steatosis grading (*p* < 0.001) [102]. Vu et al. suggested that MRI-PDFF quantification methods should sample each liver segment in both lobes and include a total surface area of ≥ 5 cm^2^ to provide a close estimate of the mean liver PDFF [103].

The MRI-determined PDFF is correlated with the histologically determined steatosis grade in patients with NAFLD. Among the studies using the MRI-based PDFF, the AUROC for diagnosis of grade > 1, grade > 2, and stage 3 ranged from 0.960 to 0.990, 0.825 to 0.90, and 0.79 to 0.92, respectively. The corresponding MRI-PDFF cut-offs for mild steatosis (grade > 1) ranged from 3.5 to 8.9% with a sensitivity of 89–97% and specificity of 88–100% [52, 53, 94–96] (Table 5).

**Table 5:** Performance of proton density fat fraction compared with liver biopsy for the detection of steatosis in patients with NAFLD

Design	Comparison with controlled attenuation parameter	Steatosis grade	Cut-off value (%)	AUROC	Se	Sp	PPV	NPV	References
Cross-sectional prospective, single center (*N* = 51)	No	Grade ≥ 1	8.9	ND	ND	ND	ND	ND	Permutt et al. Aliment Pharmacol Ther. 2012 [124]
		Grade ≥ 2	16.3	ND	ND	ND	ND	ND	
		Grade 3	25.02	ND	ND	ND	ND	ND	
Cross-sectional prospective, single center(*N* = 77)	No	Grade ≥ 1	6.4	0.989	0.97	1.00	1.00	0.71	Tang et al. Radiology. 2014 [125]
		Grade ≥ 2	17.4	0.825	0.61	0.90	0.90	0.61	
		Grade 3	22.1	0.893	0.68	0.91	0.72	0.90	
Cross-sectional prospective, single center (*N* = 142)	Yes vs. VCTE (M probe)	Grade ≥ 1	5.2	0.96	0.900	0.933	0.892	0.519	Imajo et al. Gastroenterology. 2016 [62]
		Grade ≥ 2	11.3	0.90	0.789	0.841	0.845	0.784	
		Grade 3	17.1	0.79	0.737	0.810	0.632	0.953	
Cross-sectional prospective, single center (*N* = 27), Child	No	Grade ≥ 1	3.5	ND	0.890	0.880	ND	ND	Di Martino M et al. World J Gastroenterol. 2016 [126]
		Grade ≥ 2	ND	ND	ND	ND	ND	ND	
		Grade 3	ND	ND	ND	ND	ND	ND	
Cross-sectional prospective, single center(*N* = 104)	Yes vs. VCTE (M and XL probe)	Grade ≥ 1	3.71	0.99	0.958	1.000	1.000	0.700	Park et al. Gastroenterology. 2017 [63]
		Grade ≥ 2	13.03	0.90	0.800	0.833	0.750	0.870	
		Grade 3	16.37	0.92	0.818	0.836	0.450	0.966	

*AUROC* Area under the receiver-operating characteristic, *NPV* negative predictive value, *PPV* positive predictive value, *Se* sensitivity, *Sp* specificity

### Comparison of diagnostic accuracy for steatosis grading between VCTE-CAP and MRI-PDFF in patients with NAFLD

By direct comparison, Imajo et al. demonstrated that MRI-PDFF has higher accuracy than VCTE-based CAP for diagnosing steatosis in patients with NAFLD [62]. However, they assessed VCTE using the M probe only. More recently, using a well-characterized prospective cohort of adults in the USA with biopsy-proven NAFLD, Park et al. compared the accuracy of VCTE-based CAP using both M and XL probes versus MRI-PDFF for diagnosing steatosis in patients with NAFLD. They demonstrated that MRI-PDFF was superior to CAP using M and XL probes for diagnosing steatosis in patients with NAFLD [63].

### Benefits and limitations of MRE and MRI-PDFF

MRE and PDFF methods have higher diagnostic performance for noninvasive detection of liver fibrosis and steatosis in patients with NAFLD compared with other noninvasive methods. A comparison between US-based elastography and MRE is shown in Table 6. One of the benefits of MRE is that it allows for much larger sampling compared with US techniques and liver biopsy, which may reduce sampling variability secondary to heterogeneity of fibrosis. In addition, it has been proven that MRE generally provides more reliable measurements and has lower failure rates in patients with obesity or ascites. In a recent retrospective review of a large series of 1377 MRE cases from the Mayo Clinic, the reported failure rate was < 6%, with no effect of BMI on the failure rate [104]. MRE may also be a better candidate than US-based elastography for assessing patients’ responses to new therapies for NASH.

**Table 6:** Comparison between US elastography and MR elastography

	US elastography	MR elastography
Sampling volume of liver	Little	Much^a^
HCC screening	Possible (except TE)	Good^a^
Convenience of use	Good^a^	Poor
Inter-operator reproducibility	Good ICC; TE 0.98, ARFI 0.81, SWE 0.88	Good ICC; 0.99
Intra-operator reproducibility	Good ICC; TE 0.98, ARFI 0.81, SWE 0.88	Good ICC;
Evaluation of liver fat accumulation	Available using only TE-based CAP but the diagnostic accuracy is insufficient	Available using PDFF good^a^
Ascites	Available if ascites is a little (except TE)	Available if ascites is a little good^a^
Obesity	Possible for ARFI, SWE, TE by XL probe	Good^a^
Measurements of iron deposition	Not available	Available^a^
Effect of iron overload on liver stiffness and liver fat accumulation	No effect^a^	Effect
Contraindications	No^a^	Biocompatible metal pregnancy
Cost	Low^a^	High
Available institutions	Many^a^	Not so many

^a^ Benefit

*AUROC* Area under the receiver-operating characteristic, *ARFI* acoustic radiation force impulse, *CAP* controlled attenuation parameter, *HCC* hepatocellular carcinoma, *NPV* negative predictive value, *PPV* positive predictive value, *Se* sensitivity, *Sp* specificity, *SWE* shear wave elastography, *TE* transient elastography, *PDFF* proton density fat fraction

Limitations of MRE include the possibility of failure in patients with iron overload (using a gradient echo sequence), cost, availability, and possible contraindications in patients with articles such as metallic splinters, vascular clips, and cochlear implants (Table 6). In addition, liver stiffness obtained by MRE may be influenced by extrahepatic cholestasis and acute liver injury [105, 106]. However, all major vendors now propose MRE capabilities, and new sequences such as echoplanar imaging have been shown to decrease the failure rate in the presence of hepatic iron deposition.

## HCC

The surveillance system for HCC in patients with NAFLD is problematic. Even in Japan, the estimated number of patients with NAFLD is around 10–20 million. In one study, the incidence of HCC among patients with NAFLD was 0.44 per 1000 person-years (range, 0.29–0.66), and that in NASH was 5.29 per 1000 person-years (range, 0.75–37.56) [107]. Thus, the incidence of HCC is considerably low, and it is impossible and uneconomical to perform imaging examinations for HCC surveillance in all patients with NAFLD.

The risk factors for HCC in patients with NAFLD are important to surveillance systems. The most important risk factor of developing HCC is advanced fibrosis (fibrosis stage 3 and 4). PLT count of < 19.2 × 10^4^/µL and serum hyaluronic acid level of > 42 ng/mL are considered to be good predictive markers of advanced fibrosis [27, 108]. As previously stated, several reports have recommended liver stiffness of > 9 kPa as measured by the VCTE as the cut-off value for grade 3 fibrosis in patients with NAFLD [109]. Therefore, if the PLT count is < 19.0 × 10^4^/µL, hyaluronic acid level is > 40 ng/mL, or liver stiffness is > 9 kPa as measured by the VCTE, advanced fibrosis is suspected. In addition, Kawamura et al. reported that the risk factors for HCC were advanced fibrosis, male sex, old age, and diabetes [110]. Kodama and Tokushige reported a high gamma-glutamyl transferase level in addition to the above-mentioned factors [111, 112]. Ashca et al. reported past or social drinking as a risk factor [113]. Such patients should undergo US and/or other examinations every 6 months because these patients have an elevated risk of both HCC and cardiovascular events [114].

About 85% of NAFLD-HCC developed from NAFLD with advanced fibrosis (grade > 3) in female patients with NAFLD-HCC, whereas about 60% of HCC developed from NAFLD with advanced fibrosis in male patients. In general, patients with mild fibrosis were older age and had poor control of diabetes in hepatitis C [115]. Therefore, male patients with NAFLD who are older and have poorly controlled diabetes should be taken for adequate surveillance of HCC, even if their hepatic fibrosis is not advanced. At the present time, surveillance algorithms for HCC have already been described in guidelines published by the AASLD) the European Association for the Study of the Liver and the European Organization for Research and Treatment of Cancer (EASL-EORTC) and the Japan Society of Hepatology (JSH). The JSH algorithm considered the functional imaging techniques of gadolinium ethoxybenzyl diethylenetriamine pentaacetic acid-enhanced MRI (EOB-MRI) and Sonazoid contrast-enhanced ultrasound (CEUS) to be very important diagnostic modalities, bet the AASLD and EASL-EORTC algorithms suggest that a diagnostic be made based solely on hemodynamic findings using dynamic CT/MRI and biopsy findings [116].

## Summary

Staging of liver fibrosis is essential in determining the prognosis and optimal treatment for patients with NAFLD and in guiding surveillance for the development of HCC. Several epidemiological studies have revealed that cardiovascular disease is the most common cause of death in patients with NAFLD independent from other metabolic comorbidities and that liver-related mortality is the second or third most common cause of death [24, 117–119]. Notably, histological liver fibrosis is the only independent predictive factor for the overall long-term outcome of NAFLD, including cardiovascular disease and liver-related mortality. Liver biopsy is recommended as the gold standard method for the diagnosis and staging of fibrosis in patients with NAFLD as well as other chronic liver diseases [120]. However, this procedure is associated with a risk of complications, and is costly and time-consuming for both providers and patients [8]. Furthermore, it is impossible to enforce liver biopsy in all patients with NAFLD because the estimated number of such patients has reached 80–100 million in the USA and 10–20 million or even more in Japan [121]. In this review, we have introduced several noninvasive diagnostic methods for NAFLD with a particular focus on liver fibrosis and steatosis as well as liver biopsy.

**Fig. 2:**
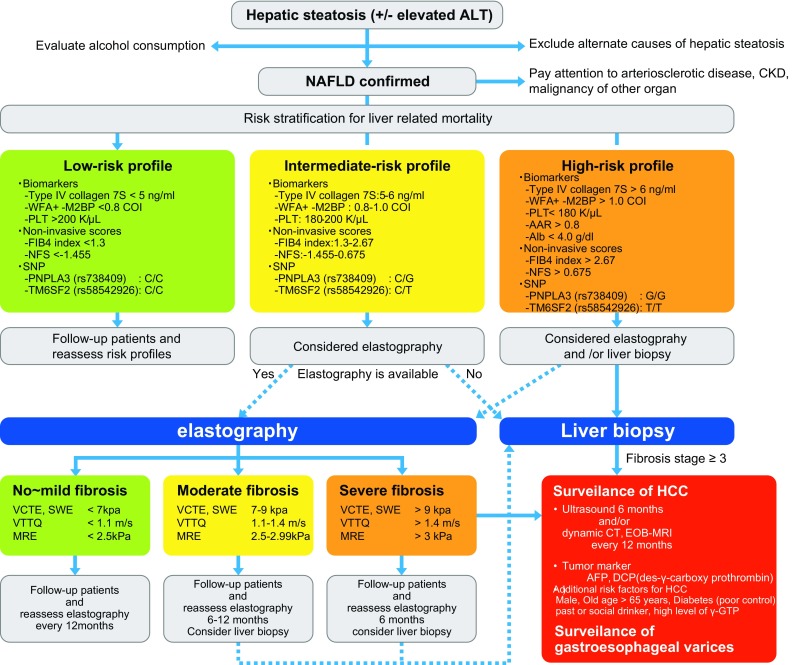
Clinical algorithm for diagnosing and following the patients with NAFLD based on liver biopsy and noninvasive methods. Solid arrow: recommended flow with consensus. Dotted arrow: recommended flow

Progression of chronic liver disease is often asymptomatic; however, patients usually present with complications at advanced stages of the disease. Noninvasive scoring systems are considered useful for selection of patients who require further inspection of liver fibrosis. US-based elastography and MRE are now becoming more widespread because they have demonstrated higher accuracy in the diagnosis of severe fibrosis and liver cirrhosis. The major advantages of these elastography techniques compared with liver biopsy are that they are painless and rapid, have no associated complications, and are universally accepted by patients. Additionally, ARFI elastography, SWE, and MRE can be performed during standard examinations of the liver that are routinely performed for HCC surveillance in patients with chronic liver disease (Table 2). Even when the diagnosis of cirrhosis is obvious, there are two additional benefits of LSM in these patients. First, a higher degree of stiffness in patients with cirrhosis could be of diagnostic value for detecting the presence of large varices [122]. Second, greater stiffness may be predictive of other complications and subsequent liver-related death [123]. In this regard, we proposed a clinical algorithm for diagnosing and following the patients with NAFLD based on liver biopsy and noninvasive methods such as scoring systems, US-based elastography and MRE, and appropriate surveillance of HCC (Fig. 2).

## Abbreviations

AAR: Aspartate aminotransferase-to-alanine aminotransferase ratio
AASLD: American Association for the Study of Liver Diseases
AGEs: Advanced glycation end products
ARFI: Acoustic radiation force impulse
BMI: Body mass index
FIB-4: Fibrosis-4
HCC: Hepatocellular carcinoma
ICC: Intra-class correlation coefficient
NAFL: Nonalcoholic fatty liver
NAFLD: Nonalcoholic fatty liver disease
NASH: Nonalcoholic steatohepatitis
MRI: Magnetic resonance imaging
NAS: NAFLD activity score
NFS: NAFLD fibrosis score
PLT: Platelet
PNPLA3: Patatin-like phospholipase domain-containing protein 3 gene
SWE: Shear wave elastography
US: Ultrasound
VCTE: Vibration-controlled transient elastography
